# Selective Pressure by Rifampicin Modulates Mutation Rates and Evolutionary Trajectories of Mycobacterial Genomes

**DOI:** 10.1128/spectrum.01017-23

**Published:** 2023-07-12

**Authors:** E. Cebrián-Sastre, A. Chiner-Oms, R. Torres-Pérez, I. Comas, J. C. Oliveros, J. Blázquez, A. Castañeda-García

**Affiliations:** a Departamento de Biotecnología Microbiana, Centro Nacional de Biotecnología (CNB), CSIC, Madrid, Spain; b Instituto de Biomedicina de Valencia (IBV), CSIC, Valencia, Spain; c Servicio de Bioinformática para Genómica y Proteómica. Centro Nacional de Biotecnología (CNB), CSIC, Madrid, Spain; d Centro Nacional de Microbiología, Instituto de Salud Carlos III (CNM-ISCIII), Majadahonda (Madrid), Spain; University of Pittsburgh

**Keywords:** DNA repair, *Mycobacterium*, *Mycobacterium smegmatis*, antibiotic resistance, drug resistance evolution, evolution, experimental evolution, mutation, mutation accumulation, rifampicin

## Abstract

Resistance to the frontline antibiotic rifampicin constitutes a challenge to the treatment and control of tuberculosis. Here, we analyzed the mutational landscape of Mycobacterium smegmatis during long-term evolution with increasing concentrations of rifampicin, using a mutation accumulation assay combined with whole-genome sequencing. Antibiotic treatment enhanced the acquisition of mutations, doubling the genome-wide mutation rate of the wild-type cells. While antibiotic exposure led to extinction of almost all wild-type lines, the hypermutable phenotype of the Δ*nucS* mutant strain (noncanonical mismatch repair deficient) provided an efficient response to the antibiotic, leading to high rates of survival. This adaptative advantage resulted in the emergence of higher levels of rifampicin resistance, an accelerated acquisition of drug resistance mutations in *rpoB* (β RNA polymerase), and a wider diversity of evolutionary pathways that led to drug resistance. Finally, this approach revealed a subset of adaptive genes under positive selection with rifampicin that could be associated with the development of antibiotic resistance.

**IMPORTANCE** Rifampicin is the most important first-line antibiotic against mycobacterial infections, including tuberculosis, one of the top causes of death worldwide. Acquisition of rifampicin resistance constitutes a major global public health problem that makes the control of the disease challenging. Here, we performed an experimental evolution assay under antibiotic selection to analyze the response and adaptation of mycobacteria, leading to the acquisition of rifampicin resistance. This approach explored the total number of mutations that arose in the mycobacterial genomes under long-term rifampicin exposure, using whole-genome sequencing. Our results revealed the effect of rifampicin at a genomic level, identifying different mechanisms and multiple pathways leading to rifampicin resistance in mycobacteria. Moreover, this study detected that an increase in the rate of mutations led to enhanced levels of drug resistance and survival. In summary, all of these results could be useful to understand and prevent the emergence of drug-resistant isolates in mycobacterial infections.

## INTRODUCTION

The acquisition and spread of antimicrobial resistance (AMR) in pathogenic bacteria are major threats to public health. It has been estimated that, without effective health care policies to control it, AMR could contribute to 10 million deaths per year by 2050, exceeding the current mortality rate from cancer (1). Huge pressure imposed over decades by antimicrobial usage in humans has led to the evolution of genetic variants and the selection and spread of antimicrobial-resistant strains.

One of the main ways to achieve antibiotic resistance is through the acquisition of mutations in different chromosomal loci (2). Bacterial cells with higher mutation rates (mutators or hypermutators) have a higher probability of acquiring favorable mutations, including those that confer antibiotic resistance. These hypermutable strains, commonly deficient in postreplicative DNA mismatch repair (MMR), can thrive under selective conditions such as antibiotic pressure (3).

In addition to driving the selection of resistant variants, many antibiotics can also cause increases in bacterial mutagenesis rates. Bactericidal antimicrobials such as fluoroquinolones, β-lactams, antifolates, and aminoglycosides can induce responses that accelerate mutation rates, driving both the generation of genetic diversity and the selection of resistant variants (3–6). Production of reactive oxygen species (ROS) and DNA damage responses are central to the accumulation of antibiotic-induced damage, and antibiotic-induced mutagenesis mechanisms can be classified into three largely intertwined groups: induction of the production of ROS, induction of the SOS response, and activation of the RpoS-regulated response (6).

The mutagenic effect of antibiotics and the selection of hypermutable variants could be especially important for bacterial pathogens such as Mycobacterium tuberculosis, which acquire antibiotic resistance exclusively through chromosomal mutations (7). Tuberculosis (TB) is one of the top causes of death worldwide and a leading cause of death from a single infectious agent, second only to COVID-19 (8). Drug resistance in M. tuberculosis makes the control of TB more challenging and remains a major global threat to public health. Close to half a million people develop rifampicin-resistant TB (RR-TB) every year, and 78% of those cases have multidrug-resistant TB (MDR-TB), which is resistant to both rifampicin and isoniazid (9). The success rate of treatment decreases considerably in patients with drug-resistant TB (success rate under 60%, with higher rates of relapse and death), requiring longer treatment with more expensive and toxic second-line drugs (8).

Rifampicin is, along with isoniazid, the most powerful first-line antibiotic for TB. It is a bactericidal drug that acts by binding to the DNA-dependent RNA polymerase subunit β (RNAP β), encoded by the *rpoB* gene, thereby inhibiting transcription (10). Emergence of mutations in the *rpoB* gene represents the main mechanism underlying rifampicin resistance and is responsible for 90 to 95% of rifampicin-resistant M. tuberculosis isolates (11). The mechanism of resistance in the remaining rifampicin-resistant isolates remains largely unknown, although the involvement of lowered cell permeability and enhanced efflux pumps has been hypothesized (12). Interestingly, rifampicin-induced ROS formation has been observed in the pathogen (13).

Experimental evolution has been widely used to study antibiotic resistance mechanisms (14). The mutation accumulation (MA) assay is a specific type of experimental evolution with single-cell bottlenecks at each serial transfer. MA is designed to allow mutations to occur in a neutral manner without selection, and consequently all nonlethal mutations can be fixed (15). When evolving MA cells are exposed to antibiotic selection, drug-induced changes in the rate and molecular landscape of mutations can be quantitatively analyzed without the interference from other factors. Using the MA assay with different bacterial cell lines, combined with whole-genome sequencing (WGS), overcomes the limitations of classical methods for determining mutation rates (16). Indeed, we have also used this experimental approach in the absence of antibiotic to determine the mutational rate and landscape of a wild-type (WT) strain of the surrogate model Mycobacterium smegmatis and a noncanonical MMR-deficient Δ*nucS* mutant derivative with a hypermutator phenotype (17).

To date, there are few studies that have quantitatively evaluated the bacterial genome-wide mutational profile by MA/WGS after antibiotic exposure (16, 18). In this work, we performed MA/WGS analyses in several independent bacterial lines of M. smegmatis wild-type and Δ*nucS* strains under increasing concentrations of rifampicin, to characterize the rate and molecular spectrum of genomic mutations and analyze the potential role of hypermutation in response to the antibiotic. In addition, we studied the evolutionary pathways to the acquisition of rifampicin resistance in M. smegmatis, with a special emphasis on the study of the appearance of mutations in *rpoB* and the corresponding resistance level conferred over time. We also searched for candidate genes enriched with mutations that may be part of the adaptative response and/or alternative resistance mechanisms. Our results provide quantitative insight into the relationship between antibiotic selective pressure and the rate and molecular spectrum of mutations, the degree to which noncanonical MMR could be involved, and the extent to which elevated mutation rates accelerate the acquisition of drug resistance in mycobacteria.

## RESULTS

### MA experiments under rifampicin selection.

In a recent work, we determined mutation rates and mutational spectra of M. smegmatis wild-type and *nucS*-null (Δ*nucS*) mutant strains by a MA assay in the absence of antibiotic (17). Once mutation rates without antibiotic have been characterized in depth, it is critical to analyze the effect of antibiotic selective pressure in the mutational landscape of mycobacteria. In this study, we have evaluated the effect of the frontline antibiotic rifampicin on mycobacterial evolution by MA experiments and WGS using M. smegmatis as a model (wild-type and Δ*nucS* strains) (see Materials and Methods). First, the MIC of rifampicin was determined for both strains in solid media. The parental strains showed identical susceptibility to the drug, each with a MIC value of 2 μg mL^−1^.

**FIG 1:**
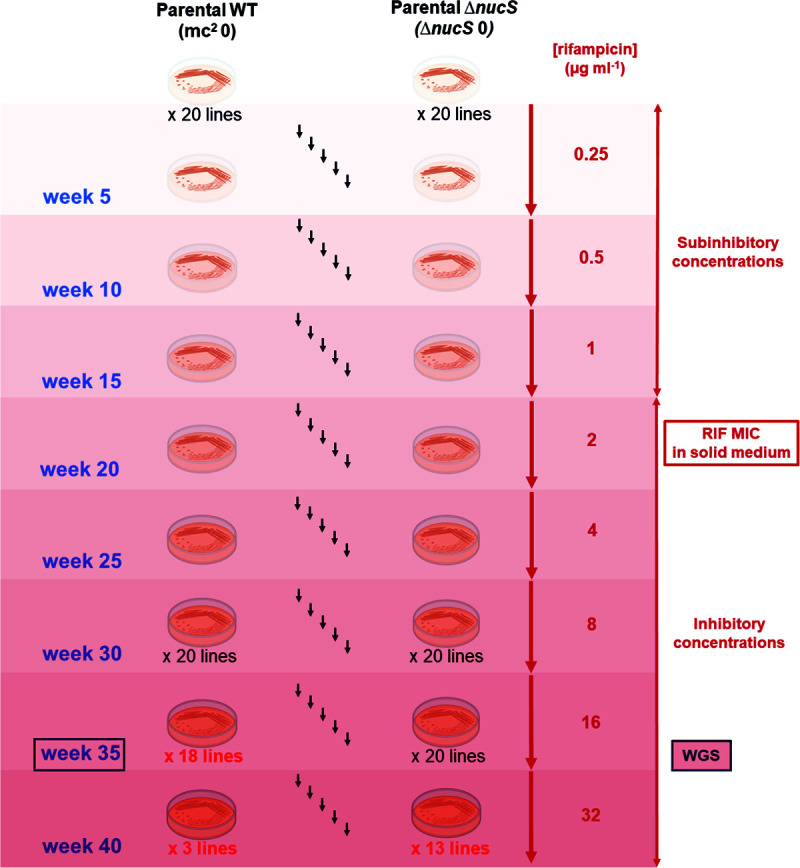
MA experimental evolution with increasing concentrations of rifampicin. The MA assay comprised 40 independent lines, generated from the M. smegmatis wild type and its *nucS*-null (Δ*nucS*) mutant derivative (20 each) and evolved in parallel with increasing antibiotic concentrations. The experimental evolution was carried out for 40 weeks, from 0.25 μg mL^−1^ rifampicin (subinhibitory concentration) until 32 μg mL^−1^ rifampicin (inhibitory concentration), doubling the concentration every 5 weeks. MA lines able to grow at week 35 with 16 μg mL^−1^ rifampicin (18 wild type-derived and 20 Δ*nucS* mutant-derived) were sequenced by WGS. The figure also shows the number of lines that survived at each rifampicin concentration (below the plates) and the number of passages (black arrows). Created with BioRender.com.

The MA assay under antibiotic pressure was performed in parallel at the same time to that in the absence of antibiotic, but rifampicin concentrations were steadily increased throughout the experiment (from 0.25 μg mL^−1^ to 32 μg mL^−1^, doubling every 5 weeks) (Fig. 1). Both experimental evolutions were carried out following the same experimental design and procedure, under the same biological conditions (growth parameters, media, and incubation time), to compare the results of both assays. Experimental evolution with rifampicin was carried out with a total of 20 lines derived from each M. smegmatis parental strain (wild type and Δ*nucS* mutant) for 40 weeks, by transferring a single random colony to a new plate every 7 days (Fig. 1). This strict bottleneck strongly restricted selection, ensuring that each emerged mutation was retained.

At the end of the experiment, by week 40, only 3 of 20 evolved wild-type lines were able to survive to the highest rifampicin concentration (32 μg mL^−1^), while the majority of the Δ*nucS* lines (13 of 20), survived. Most of the MA lines (18 of 20 wild-type and all 20 Δ*nucS* lines) survived by week 35 in the presence of up to16 μg mL^−1^ rifampicin (Fig. 1). The extinction of 85% of the wild-type lines but only 35% of *nucS*-deficient lines upon exposure to rifampicin highlights the importance of hypermutability conferred by noncanonical MMR deficiency for resistance to rifampicin in M. smegmatis.

### Mutation rates and mutational signatures under antibiotic pressure.

Mutation rates in the evolved wild-type and Δ*nucS* lines were analyzed by WGS to detect the total number of mutations in each strain. The 18 MA wild-type lines and 20 MA Δ*nucS* lines that were able to grow with 16 μg mL^−1^ rifampicin by week 35 were selected for mutational analysis. This antibiotic value is a critical point that allowed us to obtain a full set of WGS data of both strains. Genomic sequences generated by WGS were compared to the M. smegmatis mc^2^ 155 reference genome (NC_018289.1) to filter all base pair substitutions (BPSs) and small insertion/deletions (indels) found in each MA line (see Materials and Methods).

Mutation rates per genome (or nucleotide) per generation (Table 1; see Tables S1 and S2 in the supplemental material) were determined by measuring the total number of mutations in the whole genome in relation to the number of cell divisions or generations of each line (see Materials and Methods). WGS analysis identified 148 mutations in MA wild-type lines, with 0.009 mutation per genome per division (13.47 × 10^−10^ mutations per nucleotide per generation). A total number of 3,181 mutations were detected in Δ*nucS* lines, corresponding to 0.174 mutation per genome per division (258.15 × 10^−10^ mutations per nucleotide per generation). Therefore, the inactivation of noncanonical MMR in the Δ*nucS* lines prompted an ~19-fold increase in the mutation rate of M. smegmatis under rifampicin selection.

**TABLE 1:** Rates of mutation identified in the MA experiment with rifampicin

	mc^2^ 155 (WT)			Δ*nucS* mutant		
	No. lines	Total generations	Generations per line	No. lines	Total generations	Generations per line
	18	16,308	906	20	18,315	916
Mutation type	No. mutations	Mutation rate/genome/generation (×10^−3^)^*a*^^*b*^	Mutation rate/nt/generation (×10^−10^)^*a*^^*b*^	No. mutations	Mutation rate/genome/generation (×10^−3^)^*a*^^*b*^	Mutation rate/nt/generation (×10^−10^)^*a*^^*b*^
Total	148	9.07 ± 1.35	13.47 ± 2.00	3,181	173.73 ± 10.69	258.15 ± 15.95
BPSs	100	6.13 ± 1.35	9.11 ± 2.01	3,141	171.55 ± 10.54	254.93 ± 15.73
Indels	48	2.94 ± 0.70	4.36 ± 1.03	40	2.17 ± 0.68	3.23 ± 1.00

a The mutation rate per genome or nucleotide per generation ±95% confidence interval (CI) is shown.

b Statistically significant differences were observed between the WT and Δ*nucS* strains for the total mutation rate (*P* < 0.001; *t* test) and for the BPS rate (*P* < 0.001; *t* test). No statistically significant differences were observed between WT and Δ*nucS* strains for the indel rate (*P* > 0.05 by *t* test and *P* = 0.05 by Mann-Whitney U test).

**FIG 2:**
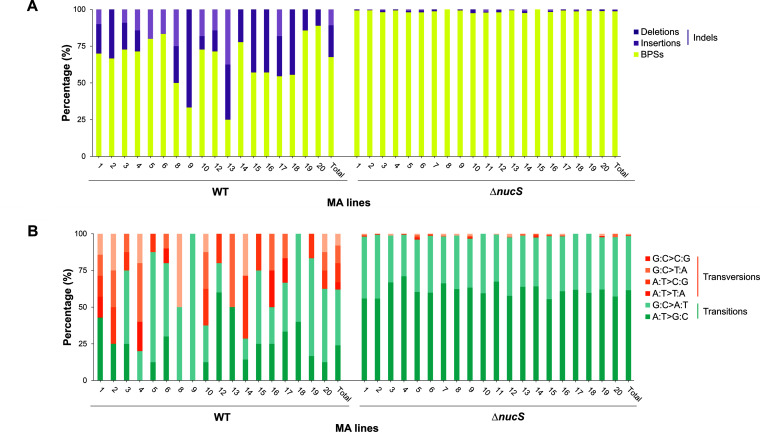
Mutational signature of the MA lines evolved with rifampicin. (A) Proportion of BPSs, deletions, and insertions in the MA lines (wild type and Δ*nucS* mutant). Percentages were calculated with respect to the total number of mutations obtained by WGS. The Mann-Whitney U test was used to compare the percentages of each mutation type between strains for BPSs (*P* < 0.001) and indels (*P* < 0.001). (B) Relative proportion of the six types of BPSs in the MA lines (wild type and Δ*nucS* mutant). Percentages were calculated with respect to the total number of BPSs obtained by WGS. The Mann-Whitney U test was used to compare the percentages of each mutation type between strains for transitions (*P* < 0.001) and transversions (*P* < 0.001). Bars are divided in portions with different colors according to the type of mutation, as follows: BPSs in yellow, indels in purple, transitions in greeny blue, and transversions in orange.

A total of 100 bp substitutions (BPSs) and 48 small insertions or deletions (indels) were detected in the evolved wild-type lines, corresponding to 9.11 × 10^−10^ BPSs and 4.36 × 10^−10^ indels per nucleotide per generation (Table 1; Table S1). The mutational signature reflects that while the majority of mutations were BPSs (with more transitions than transversions), one-third of all detected mutations were indels in the wild-type lines (Fig. 2).

In the evolved Δ*nucS* lines, the number of identified BPSs was very high, with a total of 3,141 BPSs, corresponding to 254.93 × 10^−10^ mutations per nucleotide per generation, while only 40 indels, corresponding to 3.23 × 10^−10^ indels per nucleotide per generation, were detected (Table 1; Table S2). BPSs (almost all transitions) vastly outnumbered indels in evolved Δ*nucS* lines (Fig. 2), driving the strong increase in the global mutation rate found in Δ*nucS* genomes, as previously observed during drug-free evolution (17). Furthermore, across the Δ*nucS* lines tested, the BPS rate was 28-fold higher than the wild-type one under rifampicin selection, while no significant change was observed in indel rate. All of these results together revealed that the rise in the mutation rate generated by DNA repair deficiency in the Δ*nucS* strain occurred similarly and with the same mutational pattern during drug-free evolution (17) and in the evolution under selective antibiotic pressure.

### Rifampicin effect on mutation rates: a comparison in the presence and absence of antibiotic.

**FIG 3:**
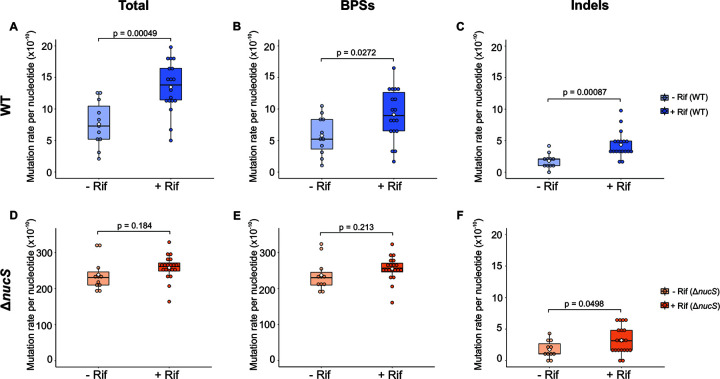
Mutation rates (total mutations, BPSs, and indels) in the presence and absence of rifampicin. Box plots show the comparison between mutation rates of MA lines evolved with rifampicin (+Rif) (this study) and without antibiotic (−Rif) (17). The rates of total mutations, BPSs, and indels are shown for the wild-type (A to C) (−Rif, light blue; +Rif, dark blue) and Δ*nucS* mutant (D to F) (−Rif, light orange; +Rif, dark orange) strains, respectively. Dots represent the mutation rate of each independent MA line. The mean of each mutation rate is indicated with a white diamond. *P* values (*t* test) are shown for each graph. *t* tests were complemented with Mann-Whitney U tests (see Table S5), as data for some types of mutations are nonparametric, although they are close to normality.

**FIG 4:**
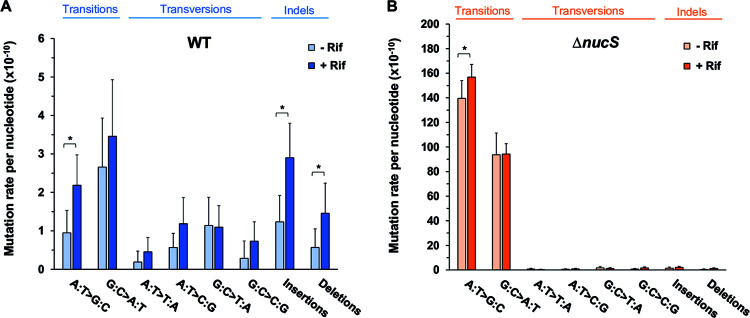
Comparison of the mutational spectra of the MA lines evolved in the presence and absence of rifampicin. (A) Mutational spectra for M. smegmatis wild-type lines evolved with rifampicin (dark blue) and without antibiotic (light blue); (B) mutational spectra for M. smegmatis Δ*nucS* lines evolved in the presence (dark orange) and absence of rifampicin (light orange). Bars represent the mutation rate per nucleotide per generation for each type of DNA mutation obtained by WGS data of MA experiments from this study and our previous work (17). Error bars indicate 95% confidence intervals (CIs). *, *P* < 0.05 (*t* test). *t* tests were complemented with Mann-Whitney U tests (see Table S5), as data for some types of mutations are nonparametric, although they are close to normality.

To determine the effect of rifampicin on the overall mutation rates in this study, we compared the evolution of M. smegmatis MA lines with and without (17) selective pressure (Fig. 3 and 4). A complete data set of the WGS results (with and without antibiotic) is included in Tables S1 to S5.

Antibiotic selection led to a doubling in the mutation rate of the wild-type strain (mutation rate per genome per generation of 0.009 with rifampicin versus 0.005 without; mutation rate per nucleotide per generation of 13.47 × 10^−10^ versus 7.58 × 10^−10^, respectively) (Fig. 3A). No significant effect on the already high mutation rate of the Δ*nucS* strain was found in the evolved *nucS*-deficient strain with rifampicin (mutation rate per genome per generation of 0.174 with rifampicin versus 0.166 without and mutation rate per nucleotide per generation of 258.15 × 10^−10^ versus 238.53 × 10^−10^, respectively) (Fig. 3D).

To dissect how rifampicin could promote the emergence of mutations across the genomes, we analyzed the resulting mutational spectrum after experimental evolution in the presence and absence of rifampicin (Fig. 3 and 4; and Tables S1 to S5). In the wild-type lines, the rise in the mutation rate was generated by an increase in both rates of BPSs and indels (Fig. 3B and C). Although rates of almost all types of mutations were higher in the wild-type lines grown with rifampicin, only A:T > G:C transitions, insertions, and deletions were significantly increased (2.3-, 2.1-, and 3.7-fold higher with rifampicin than without rifampicin, respectively) (Fig. 4A). The sum of all of these mutations represents almost half of the total mutations detected in the wild-type lines evolved with rifampicin, but less than one-third under antibiotic-free evolution. In the Δ*nucS* strain, the overall mutation rate was not significantly increased under rifampicin selection (Fig. 3D), although BPS and indel rates were slightly higher (Fig. 3E and F). In this sense, the very high mutation rate of this strain could mask any further effect of the antibiotic. Nevertheless, in Δ*nucS* lines, rifampicin selection resulted in a one-tenth increase in the A:T > G:C transition, a type of mutation specially increased by *nucS* inactivation (Fig. 4B). A moderate increase for insertions and deletions in Δ*nucS* lines was also found (Tables S1 to S5), although the contribution of indels to the overall Δ*nucS* mutation rate was very small (Fig. 4B).

In summary, this study showed that rifampicin selective pressure had an effect on mutation rates of the M. smegmatis wild type. Although the DNA repair deficiency due to *nucS* inactivation could have made the Δ*nucS* lines more susceptible to rifampicin-induced mutations, rifampicin selection had a major effect on the mutation rates in the wild-type lines.

### Evolution of the levels of rifampicin resistance under antibiotic selective pressure.

To understand how the drug resistance levels of the MA lines evolved to acquire rifampicin resistance, the susceptibility to rifampicin of all of the MA lines obtained in this study was measured. First, we determined MIC values of rifampicin for each MA line at weeks 0, 10, 20, 25, 30, 35, and 40 (see Materials and Methods). Both wild-type and Δ*nucS* parental lines were slightly more susceptible to rifampicin in liquid cultures (MIC in 7H9 broth of 1 μg mL^−1^ for both parental strains) than in agar plates (MIC in 7H10 agar of 2 μg mL^−1^ for both strains).

**FIG 5:**
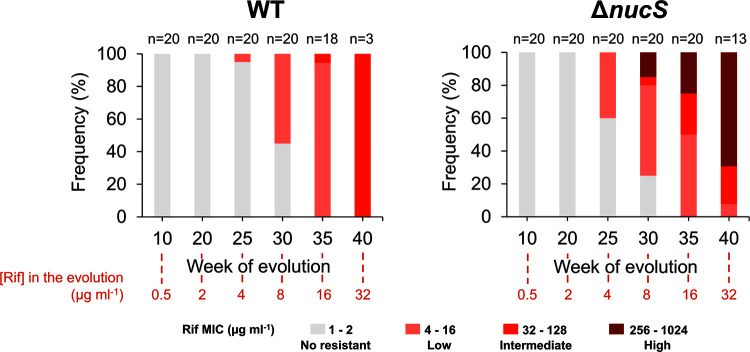
Levels of resistance to rifampicin in the MA lines during experimental evolution under antibiotic selection. The frequency (percentage) of wild-type (left) and Δ*nucS* mutant (right) lines with different levels of resistance to rifampicin through the time (in weeks) is shown. Percentages were calculated with respect to the total number of surviving lines in each week (*n*). The MIC values used to define the levels of rifampicin resistance were as follows: no resistance, 1 to 2 μg mL^−1^ (gray); low resistance, 4 to 16 μg mL^−1^ (light red); intermediate resistance, 32 to 128 μg mL^−1^ (medium red); and high resistance, 256 to 1,024 μg mL^−1^ (dark red). The numbers shown in red below the graphs indicate the rifampicin concentration used at the corresponding week. Statistically significant differences were observed in the distribution of resistance levels between strains (*P* < 0.001; Pearson’s χ^2^).

Analysis of the gradual increase in rifampicin resistance during MA evolution is shown in Fig. 5. Three levels of drug resistance were defined according to the ranges of rifampicin MIC values: low resistance (MIC of 4 to 16 μg mL^−1^), intermediate resistance (MIC of 32 to 128 μg mL^−1^) and high resistance (MIC of 256 to 1,024 μg mL^−1^). When both the wild-type and Δ*nucS* lines were exposed to subinhibitory concentrations of rifampicin (<2 μg mL^−1^), evolved MA lines did not acquire rifampicin resistance and MIC values remained similar to those observed in parental strains (Fig. 5). In both strains, the first lines that acquired rifampicin resistance (with a significant increase in MIC) emerged at week 25, under inhibitory concentrations of antibiotic (≥2 μg mL^−1^) (Fig. 5). From this point forward, under increasing selective pressure, the proportion of resistant lines increased progressively in both strains, even though the proportion of resistant lines (at weeks 25 and 30) was higher in Δ*nucS* lines than in wild-type lines (Fig. 5). When the resistance levels of the MA lines were examined in both strains, we found a differential response of each strain to the antibiotic pressure (Fig. 5). During the final stages of evolution (week 30 onwards), Δ*nucS* lines were able to acquire intermediate to high levels of rifampicin resistance and the majority of highly resistant lines were among the surviving lines by the end of the evolution. In contrast, wild-type lines acquired low levels of rifampicin resistance and only a few lines survived and were able to reach intermediate levels of antibiotic resistance by the end of experimental evolution.

### Acquisition of *rpoB* mutations in wild-type and Δ*nucS* lines under rifampicin selection.

The appearance of mutations in the *rpoB* gene, most of them at the rifampicin resistance determining region (RRDR), constitutes the main cause of rifampicin resistance in bacteria, including mycobacteria (19, 20). Considering the important role of *rpoB* mutations in the acquisition of rifampicin resistance, we analyzed the *rpoB* sequence from all of the MA lines (weeks 0, 10, 20, 25, 30, 35, and 40) during their evolution, in order to assess correlations with the development of antimicrobial resistance.

**FIG 6:**
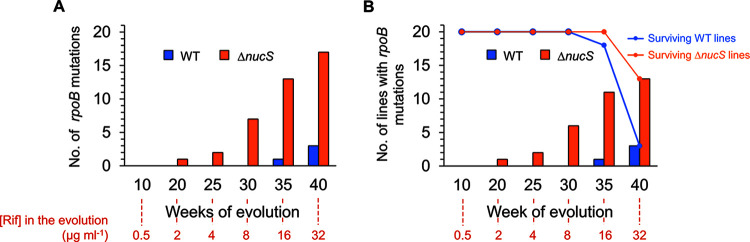
Acquisition of *rpoB* mutations in the MA lines evolved with rifampicin. (A) Dynamics of the total number of *rpoB* mutations in the MA experiment. Bars show the total number of *rpoB* mutations that accumulated all of the MA lines through the time. (B) Dynamics of the total number of lines with *rpoB* mutations in the MA experiment. Bars represent the number of lines containing *rpoB* mutations though the time (in weeks). Dots of the line plot represent the number of surviving lines (wild type, blue; Δ*nucS* mutant, orange). The numbers shown in red below the graphs indicate the rifampicin concentration used at the corresponding week. Statistically significant differences were observed in the distribution of lines with *rpoB* mutations between strains (*P* < 0.001; Pearson’s χ^2^).

At the end of experimental evolution, a total of 20 point mutations were detected in *rpoB*, with three in the wild-type-derived lines and 17 in the Δ*nucS* mutant-derived lines (Fig. 6A; Tables S6 and S7). These data suggest that hypermutability (conferred by noncanonical mismatch repair deficiency) facilitated the acquisition and fixation of a higher number of *rpoB* mutations in Δ*nucS* lines compared with wild-type-derived lines during experimental evolution under antibiotic pressure. We observed one transition and two transversions in *rpoB* in the wild-type-derived lines, while all mutations found in *rpoB* of Δ*nucS* lines were transitions, demonstrating that inactivation of *nucS* led to an increase in the number of transitions (Tables S6 and S7) (17, 21).

When the dynamics of *rpoB* mutation appearance was assessed (Fig. 6A), we found that all *rpoB* mutations arose under inhibitory concentrations of rifampicin and most of them during the final steps of the evolution when the cell lines were exposed to high concentrations of antibiotic. While the wild-type strain did not acquire any *rpoB* mutations until week 35 (16 μg mL^−1^ rifampicin), *rpoB* mutations appeared earlier in the hypermutable *nucS*-deficient strain, with the first detected at weeks 20 to 25, one-third arising by week 30, and then the number increasing through weeks 35 to 40. Therefore, the absence of *nucS* favored a more rapid acquisition of *rpoB* mutations during experimental evolution under rifampicin selection.

A strong association between the acquisition of *rpoB* mutations and enhanced survival on rifampicin was observed (Fig. 6B). Only evolved lines harboring *rpoB* mutations survived under selection with 32 μg mL^−1^ rifampicin at the end of the evolution (week 40). As a result, *rpoB* mutations were observed in all the three wild-type-derived lines, while all the remaining 13 Δ*nucS*-derived lines also had mutations in *rpoB*. Only two Δ*nucS* lines harboring *rpoB* mutation did not survive until week 40.

### Analysis of the levels of rifampicin resistance conferred by *rpoB* mutations.

**FIG 7:**
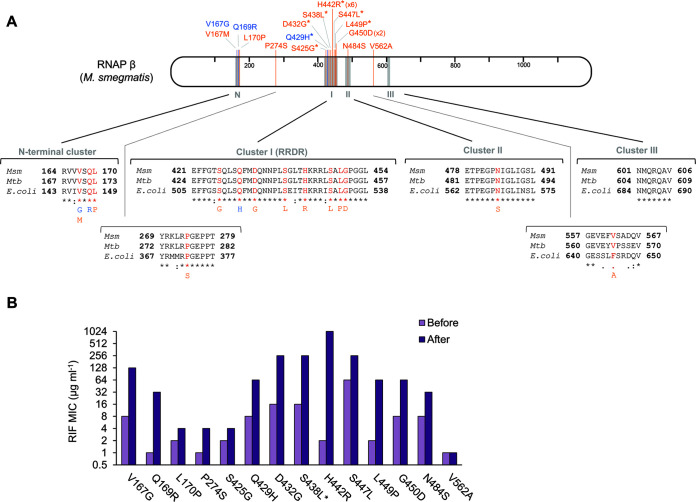
Mutations in *rpoB* in the MA lines: identification and effect on antibiotic resistance. (A) Types of RNAP β substitutions generated by *rpoB* mutations. M. smegmatis RNAP β is represented with amino acid numbering. Gray regions indicate the rifampicin resistance clusters described in E. coli (20, 23). Amino acid substitutions generated by *rpoB* mutations in the MA lines are shown in blue (wild-type lines) and orange (Δ*nucS* mutant lines). Mutations associated with resistance in M. tuberculosis by the WHO catalogue (26) are marked with asterisks. Below are shown protein alignments of RNAP β of M. smegmatis (*Msm*), M. tuberculosis (*Mtb*), and E. coli, with the amino acid positions where mutations were detected (red). (B) Effect of RNAP β substitutions on rifampicin resistance. Bars show the rifampicin MIC of the MA lines before (light purple) and after (dark purple) the acquisition of each *rpoB* mutation. When the *rpoB* mutation was present in more than one line (H442R and G450D), a representative example is shown. *, S438L substitution appeared together with V167M at the same week of the evolution.

Analysis of the identity of the emerged *rpoB* mutations detected a total of 16 different mutated positions (codons): 15 nonsynonymous mutations and one synonymous mutation. As a result, a wide diversity of amino acid substitutions was generated in the RNAP β protein sequence in the evolved lines, with 3 different types and 12 different types of amino acid substitutions observed in the wild-type and Δ*nucS* strains, respectively (Fig. 7A).

Four different conserved clusters of RNAP β amino acid substitutions (N-terminal cluster, cluster I/RRDR, cluster II, and cluster III) have been identified in rifampicin-resistant isolates (Fig. 7A) (22, 23). In this study, most of the RNAP β substitutions found in the evolved lines were located in the RRDR (cluster I), the main region with rifampicin resistance-conferring mutations in prokaryotes (20, 24). The remaining amino acid substitutions were found scattered over different regions of the RNAP β sequence, including the N-terminal cluster, with four substitutions, and cluster II, with one substitution (Fig. 7A). The hypermutator phenotype of the Δ*nucS* strain facilitated the acquisition of a wider range of *rpoB* mutations compared with the wild-type one, with several RNAP β substitutions able to diversify the pathways to antibiotic resistance (see next section).

The most common RNAP β amino acid mutation found in the higher number of MA lines (six Δ*nucS* lines) was H442R. This rifampicin resistance mutation corresponds to one of the most frequent and prominent mutations found in M. tuberculosis clinical strains (H445R mutation in M. tuberculosis RNAP β), conferring a high level of drug resistance (25). Some Δ*nucS* lines also contained other well-known rifampicin resistance mutations, D432G, S438L, and S447L, all of them among prevalent drug resistance mutations in M. tuberculosis clinical strains (D435G, S441L, and S450L mutations in M. tuberculosis RNAP β) (25, 26). Interestingly, we also observed a “borderline” resistance mutation in a Δ*nucS* mutant-derived line, L449P (L452P mutation in M. tuberculosis), conferring an intermediate level of rifampicin resistance (26, 27). While Δ*nucS* lines acquired several different high-level rifampicin resistance mutations under antibiotic pressure due to the strains’s hypermutator phenotype, only one well-known rifampicin resistance mutation was detected in the RRDR of a wild-type line, namely, Q429H (corresponding to Q432H mutation in M. tuberculosis RNAP β, a high-confidence M. tuberculosis resistance mutation according to WHO criteria) (26, 28).

To evaluate the effect of the different *rpoB* mutations on the acquisition of rifampicin resistance, we compared the MIC values of the evolved lines before and after the emergence of each *rpoB* mutation (Fig. 7B). Once all *rpoB* mutations were investigated, most *rpoB* mutant lines exhibited increased MIC values for rifampicin associated with the emergence of the corresponding *rpoB* mutation. These results support a strong link between *rpoB* mutations and the acquisition of drug resistance (although the effect of some other simultaneous mutations cannot be ruled out). Most RRDR mutations were found in isolates that showed high level of rifampicin resistance (≥256 μg mL^−1^), while mutations located in clusters outside the RRDR seemed to generate low to intermediate levels of rifampicin resistance (Fig. 7B).

### Evolutionary pathways to the acquisition of rifampicin resistance.

**FIG 8:**
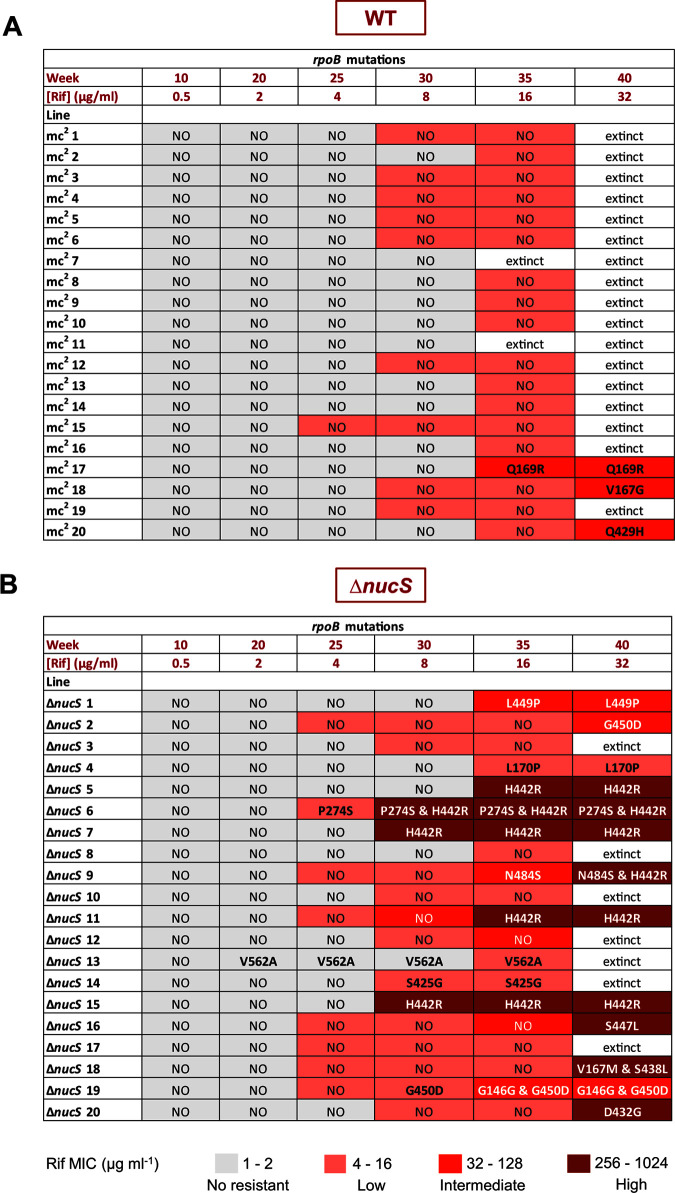
Evolutionary trajectories of the MA lines. (A) Pathways to acquire rifampicin resistance in the MA wild-type lines; (B) pathways to acquire rifampicin resistance in the MA Δ*nucS* mutant lines. The tables show the emergence of *rpoB* mutations in each MA line during the experimental evolution (in weeks). Levels of rifampicin resistance of the evolved lines are indicated according to their MIC values, with the following color code: no resistance, 1 to 2 μg mL^−1^ (gray); low resistance, 4 to 16 μg mL^−1^ (light red); intermediate resistance, 32 to 128 μg mL^−1^ (medium red); and high resistance, 256 to 1,024 μg mL^−1^ (dark red).

To reconstruct the evolutionary trajectories to rifampicin resistance, we first focused on the acquisition of rifampicin resistance by *rpoB* mutations and the corresponding MIC values (rifampicin resistance levels) from each isolate during experimental evolution (Fig. 8; Tables S6 and S7).

Investigations into the evolutionary pathways leading to rifampicin resistance in wild-type-derived lines (Fig. 8A) revealed that almost all were able to acquire low levels of drug resistance by *rpoB*-independent mechanisms, with almost half of the wild-type lines surviving at week 30 and all of the surviving lines at week 35 having low levels of resistance. From week 35, the emergence of *rpoB* mutations led to the acquisition of intermediate levels of drug resistance in three wild-type lines, allowing them to grow until the end of the evolution by week 40.

In Δ*nucS*-derived lines (Fig. 8B), we observed that the pathways leading to rifampicin resistance were more complex and diverse than the wild-type ones. The Δ*nucS* lines reached low levels of resistance earlier, by week 25. Then, most of them acquired intermediate to high rifampicin resistance levels through mutations of *rpoB*, from weeks 25 to 40, leading to a high proportion of surviving drug-resistant isolates.

When the resistance gain mutation timing and effects were analyzed, we found that under strong selective pressure (weeks 30 to 40), the number of evolved Δ*nucS* lines with high level of resistance almost doubled with increasing concentrations of antibiotic: 3 lines by week 30 at 8 μg/mL, 5 lines by week 35 at 16 μg/mL, and 9 lines by week 40 at 32 μg/mL. This effect was caused by a steady acquisition of *rpoB* mutations in the Δ*nucS* lines during the evolution (with 5 *rpoB* mutations that emerged at week 30, 6 at week 35, and 6 at week 40). The accumulation of *rpoB* mutations allowed us to explain why the majority of Δ*nucS* lines that survived exhibited high levels of rifampicin resistance.

The most frequent pathway to acquire rifampicin resistance among the Δ*nucS* lines was through RRDR mutations (Fig. 8B). The emergence of an RRDR mutation in *rpoB* often conferred high level of drug resistance from no resistance or lower resistance levels (H442R mutation observed in Δ*nucS* lines 5, 7, 11, and 15; D432G mutation in Δ*nucS* line 20; S447L mutation in Δ*nucS* line 16). In some cases, *rpoB* mutations generated intermediate levels of drug resistance in one step, as observed in the L449P mutation in Δ*nucS* line 1 or the G450D mutation in Δ*nucS* line 2. All of the Δ*nucS* lines with intermediate or high levels of rifampicin resistance through RRDR mutations were able to survive by the end of the experimental evolution. Alternatively, few Δ*nucS* lines acquired the non-RRDR *rpoB* mutation alone (L170P mutation in Δ*nucS* line 4, V562A in Δ*nucS* line 13, and S425G in Δ*nucS* line 14), although these Δ*nucS* lines had low level of resistance and, in some cases, they did not progress further.

Although a single-step progression was the most frequent pathway leading to drug resistance, the Δ*nucS* strain exhibited multiple different evolutionary trajectories, including in some cases a multistep acquisition of rifampicin resistance-conferring mutations (Fig. 8B). In two Δ*nucS* lines, Δ*nucS* 6 and 9, we initially detected low to intermediate rifampicin resistance levels by acquisition of an *rpoB* mutation outside the RRDR and then high levels of drug resistance conferred by the sequential mutation H442R in the RRDR. Another trajectory found in the Δ*nucS* lines was the simultaneous acquisition of two different *rpoB* mutations, leading to a high level of resistance in the double mutant (Δ*nucS* line 18 with mutations V167M and S438L).

Acquisition of low and even intermediate levels of rifampicin resistance was observed in some cases in the absence of any *rpoB* mutation (Fig. 8). A deeper study of the evolution of rifampicin MIC values through the time (Tables S6 and 7) revealed multiple cases where the level of antibiotic resistance increased (4-to 16-fold increase in the MIC values) without any associated *rpoB* mutation. Therefore, mutation of genes other than *rpoB* could contribute to the acquisition of rifampicin resistance in mycobacteria.

### Identification of candidate genes under positive antibiotic selection by rifampicin.

In the MA evolution, mutations in rifampicin resistance-associated genes could be subjected to positive selection by antibiotic exposure. To provide insights into the complex mechanisms underlying rifampicin resistance, genes that were enriched with mutations during evolution with rifampicin were identified. We expected to find a higher number of mutations in those genes under antibiotic selection (evolution with rifampicin) in comparison with the accumulation of random mutations (evolution without antibiotic). The use of the Δ*nucS* strain in this experimental approach was a valuable tool to detect loci under selection, as a large number of mutations accumulated across the whole M. smegmatis genome during the course of experimental evolution.

All mutations (single nucleotide polymorphisms [SNPs] and indels) detected in the evolved lines by WGS of both experimental evolution studies, with and without antibiotic, were included in the analysis to filter the genes under selection. Genes that accumulated four or more mutations under antibiotic selection and more than double the number of mutations observed in the absence of antibiotic were considered candidate genes. In addition, selected genes should have more nonsynonymous mutations than synonymous ones to be included as candidate genes. According to these criteria, 18 genes were selected with an increased number of allelic variants over the expected random ones (Table 2).

**TABLE 2:** List of candidate genes under positive selective pressure in the MA experiment with rifampicin

Gene ID (NC_018289.1)	Description	No. of mutations	
		+Rif^*a*^	−Rif^*b*^
MSMEI_1105	Amino acid permease-associated region	4	0
MSMEI_1250	NERD domain protein	4	1
MSMEI_1328 (*rpoB*)	DNA-directed RNA polymerase subunit β	14^*c*^	4
MSMEI_1418	Putative integral membrane protein	4	0
MSMEI_1810 (*selB*)	Selenocysteine-specific translation elongation factor	5	0
MSMEI_1903 (*mchK*)	Transmembrane cation transporter	8	0
MSMEI_2699	Nucleic acid binding OB-fold tRNA/helicase-type	11	1
MSMEI_2701 (*trkB*)	Trk system potassium uptake protein	4	0
MSMEI_2763 (*narB*)	Putative oxidoreductase (nitrate reductase)	5	0
MSMEI_2973 (*carB*)	Carbamoyl-phosphate synthase large chain	4	1
MSMEI_3041	Probable α-(1→6)-mannopyranosyltransferase	4	0
MSMEI_3863	HAD-superfamily hydrolase subfamily IIB	4	1
MSMEI_4371 (*dnaG*)	DNA primase	4	0
MSMEI_4625 (*mmpL*)	Transmembrane transport protein	4	0
MSMEI_5007 (*narH*)	Respiratory nitrate reductase (β chain)	4	0
MSMEI_5666 (*purL*)	Phosphoribosylformylglycinamidine synthase subunit	4	1
MSMEI_5993	Carboxymuconolactone decarboxylase	4	1
MSMEI_6055	Conserved transmembrane protein	5	1

a Data from the MA experiment with rifampicin (this work).

b Data from the MA assay in the absence of antibiotic (17).

c Thirteen *rpoB* mutations were detected by WGS and confirmed by PCR; 1 *rpoB* mutation was identified only by PCR.

The loci under selective pressure include multiple genes related to membrane transport, metabolic enzymes and some hypothetical conserved proteins with unknown functions (Table 2). Some of the candidate genes enriched in mutations could experience selection for resistance. Indeed, *rpoB* is the gene with the highest number of mutations among the selected genes, being under a strong positive selection in the assay (ratio of nonsynonymous to synonymous substitutions [*dN*/*dS*] = 4.19). In summary, the evolution with antibiotic exerted selective pressure on the M. smegmatis genome that resulted in the appearance of more mutations in specific genes with a potential role in adaptation and resistance to rifampicin. Further efforts need to be done to characterize these candidate genes and reveal their association with the development of antibiotic resistance in mycobacteria.

Finally, mutations in genes under selection could influence the mutation rates analyzed in this experimental evolution. To measure unbiased mutations, mutation rates (total, BPSs, and indels) were recalculated excluding mutations in candidate genes under selection (Table S8). They showed no substantial differences from previously calculated mutation rates (Table 1).

### Acquisition of rifampicin resistance by *rpoB*-independent mechanisms.

Although the emergence of high levels of drug resistance depends on *rpoB* mutations, the evolution with rifampicin showed that some evolved lines reached low to intermediate resistance levels in the absence of any *rpoB* mutation (see previous sections). In some cases, mutations in genes under positive selection might have a role in rifampicin resistance.

Among the candidates with positive selection (Table 2), we found that mutations in the *mchK* and *trkB* genes could be associated with antibiotic resistance during experimental evolution. In this sense, the *mchK* and *trkA* (a *trkB* homolog) genes were previously selected in a screening of a library searching for rifampicin resistance genes and led to 8- to 16-fold increases in rifampicin resistance by gene deletion (29, 30). *mchK* and *trkB* genes encode proteins that mediate K^+^ uptake into the cells: MchK is an ion channel protein and TrkB is a regulator of K^+^ uptake (as TrkA, both encoded in the same operon) (29, 30).

To provide insight into the role of ion transport in rifampicin resistance, we detected the emergence of mutations in *mchK* and *trkB* by sequencing (see Materials and Methods) and then analyzed their levels of resistance (see Fig. S1 in the supplemental material). During experimental evolution, eight MA lines (three wild-type lines and five Δ*nucS* lines) with *mchK* mutations and four Δ*nucS* lines with *trkB* mutations were detected. A correlation was detected between the emergence of some *mchK* and *trkB* mutations and increased MIC values (2-fold to 8-fold increases) (Fig. S1), while other ones had no effect in MIC values. In a few cases, they seemed to contribute to the acquisition of low levels of resistance (*mchK* mutations in wild-type lines 4, 6, and 19 and Δ*nucS* lines 2, 9, and 19; *trkB* mutation in Δ*nucS* line 20) or even an intermediate level of resistance (*trkB* mutation in Δ*nucS* line 12). The emergence of *mchK* and/or *trkB* mutations was frequently followed by a further mutation in *rpoB* in the Δ*nucS* lines (seven lines in total), leading to a high level of rifampicin resistance in these lines. The evaluation of gene-specific alterations is required to test the effect of each specific mutation on MIC values and confirm its possible role in antibiotic resistance.

These results suggest that alternative resistance mechanisms could be involved on the evolution of drug resistance in mycobacteria. A subset of genes under positive pressure could participate in the general response of mycobacteria to antibiotic-induced stress, including in some cases different pathways of drug resistance. In conclusion, our experimental approach could be a useful tool to detect loci and gene variants involved in the evolutionary pathways to antibiotic adaptation and resistance.

## DISCUSSION

MA/WGS experiments have been widely used to measure mutation rates in prokaryotes and eukaryotes (31, 32), as well as to evaluate the effect of antibiotic selection on genome-wide mutations in bacteria (14, 16, 18). In this work, the effect of long-term exposure to rifampicin was analyzed at the genome-wide level by MA/WGS in M. smegmatis. Rifampicin treatment resulted in a genome-wide mutagenic effect, with mutation rates duplicated in the wild-type strain and a weaker effect on the already hypermutator Δ*nucS* strain. While a 2-fold increase in mutation rate could be considered mild, it has been demonstrated that even modest changes in mutation frequencies can significantly influence the evolution of antibiotic resistance (33). Therefore, rifampicin exposure acts as a selective agent for potential drug resistance mutations, as well as promoting genetic diversity, thus increasing the likelihood of acquisition and fixation of mutations that confer drug resistance. It is important to note that the inhibitory concentrations of rifampicin used in this study are on the same order as the ones reported in patients (34).

Many different antibiotics have been described as promoters of genetic variation through a transient increase in mutation rates and genetic instability (35), including some that directly affect DNA replication and genome integrity (36, 37). The inhibitory effect of rifampicin on DNA expression may also contribute to generate genetic instability, as there is a strong connection between DNA replication and transcription, with specific DNA repair mechanisms involved in the resolution of replication/transcription conflicts that affect DNA integrity (38, 39). Treatments with some antibiotics (i.e., β-lactams, aminoglycosides, and quinolones) can produce increased generation of toxic ROS, resulting in higher mutation rates (5, 40) and bacterial death (41–43). Along with isoniazid and pyrazinamide (44), rifampicin induces an oxidative burst that contributes to the antibiotic-mediated killing in M. tuberculosis (13). Rifampicin could cause DNA damage through increased ROS production in M. smegmatis. It has been shown using fluorescent probes that increased ROS levels were generated in M. smegmatis after rifampicin treatment (45). Here, we also found higher ROS levels using the DCFDA (2,7-dichlorodifluorescein diacetate) probe (see Fig. S2 in the supplemental material), supporting the role of ROS as a possible agent of rifampicin-induced mutagenesis. In addition, ROS were able to improve the susceptibility to rifampicin (46, 47) and had a synergistic effect on reducing the MIC of rifampicin (48). Interestingly, enhanced ROS generation depends on the binding of the antibiotic to the RNAP, and rifampicin-dependent increases in ROS levels are not observed in resistant strains with *rpoB* mutations (13). Evolved Δ*nucS* lines, most of them with *rpoB* mutations, could be desensitized to the mutagenic effect of rifampicin during experimental evolution. Further efforts are needed to identify the mechanisms underlying antibiotic-induced mutagenesis of rifampicin in mycobacteria.

This study investigated the effect of NucS-dependent noncanonical MMR deficiency on the evolution of drug resistance mutations *in vivo* in mycobacteria and revealed that the Δ*nucS* strain (hypermutator) had a higher probability of acquiring adaptive mutations, including those that confer rifampicin resistance. Hypermutability confers evolutionary advantages for bacterial adaptation (49), by favoring the acquisition of mutations that promote the response to stressful and challenging environments such as antibiotic treatments (50–52). In Δ*nucS* lines, the mutation-driven emergence of antibiotic-resistant isolates increased rapidly and strongly, with larger number of drug resistance-conferring mutations (*rpoB* mutations) and higher levels of drug resistance than in wild-type ones. Therefore, inactivation of noncanonical MMR accelerated the appearance and fixation of drug resistance to rifampicin in mycobacteria. Not surprisingly, hypermutators are widespread in clinical isolates of multiple pathogens (53–55), where the beneficial acquisition of antibiotic resistance seems to be a key factor for their success during infection (3, 56, 57). The MA experimental approach revealed the mechanisms that could support the potential rise and spread of a *nucS*-deficient strain in a population under antibiotic pressure. In a bacterial population, indirect selection or second-order selection (a linkage between a beneficial mutation and the mutator allele), supports the selection for hypermutator clones (58, 59). In this study, the success of the hypermutator Δ*nucS* strain, with high survival rates under increasing concentration of rifampicin, correlates with the coselection of high-level resistance mutations in *rpoB*, as all surviving MA lines had at least one *rpoB* mutation.

The high evolvability (ability of an organism to generate genetic variation during evolution) of the *nucS*-deficient strain is a powerful tool for the acquisition of rifampicin resistance during evolution under antibiotic selection. Δ*nucS* lines generated a wider diversity of antibiotic resistance-conferring mutations across the *rpoB* gene than the wild-type lines, with single and even double mutations (simultaneous or successive), leading to an increased number and range of evolutionary pathways for rifampicin resistance acquisition. Since the emergence of *rpoB* mutations is frequently associated with fitness cost (60, 61) and compensatory mutations (62), this study opens the potential to search for new compensatory mutations that may relieve the loss of fitness associated with rifampicin resistance. Although several studies support the emergence and prolonged survival of hypermutators (63–65), it would be interesting to evaluate the effect of a constitutive (heritable) *nucS*-deficient strain in terms of growth and fitness during long-term experimental evolution.

The analysis of the MA evolution under antibiotic pressure provides details on the mechanisms of acquisition of rifampicin resistance. Most rifampicin-resistant M. tuberculosis strains have nonsynonymous mutations in the *rpoB* gene, the majority in the RRDR (19), preventing the effective binding of rifampicin within the RNAP β subunit (66). It is remarkable that, in this study, most of the mutations detected in the evolved MA lines were also found in the RRDR, including H442R (the most frequent one), D432G, S447L, and S450L, mutations commonly found in M. tuberculosis rifampicin-resistant isolates (25) and listed as drug resistance-conferring mutations by the WHO catalogue of M. tuberculosis mutations (26). The emergence of high-level resistance by RRDR *rpoB* mutations was the most frequent evolutionary pathway for rifampicin resistance during experimental evolution. The selection of high-level resistance mutations has occurred even under low rifampicin concentrations during this experimental evolution, as drug resistance mutations could be selected by low antibiotic pressure (67). The emergence of a specific high-level resistance mutation, H442R, in one-third of all Δ*nucS* lines revealed an interesting phenomenon of evolutionary convergence, suggesting that multiple possible pathways to rifampicin resistance seem to be modulated and restricted to some preferred ones, as previously reported (68, 69). It is also interesting to note that the mutational profile of the Δ*nucS* strain could facilitate some specific pathways to rifampicin resistance.

Additionally, different non-RRDR *rpoB* mutations and other *rpoB*-independent mutations conferring low or intermediate levels of resistance were also detected in evolved lines. Antibiotic resistance mutations associated with low and/or intermediate drug resistance are key intermediate steps in the pathways leading to high levels of resistance (70). No *rpoB* mutations in the MA lines were required to survive under subinhibitory concentrations of rifampicin. In fact, regulation of gene expression may support the growth of the MA lines under low antibiotic pressure, as rifampicin induces tolerance as an adaptative response to antibiotic stress in mycobacteria (71, 72). Beyond this point, the acquisition of one or several rifampicin resistance-conferring mutations was required for survival and the wider mutational landscape of the Δ*nucS* strain was a strong advantage in the acquisition of resistance.

Since some drug-resistant isolates lack *rpoB* mutation, alternative pathways for rifampicin resistance, involving different transporters and efflux pumps, antibiotic-modifying enzymes, and modulators of permeability, have been proposed (12). Several strategies have been used to screen for potential genes involved in drug resistance in mycobacteria, including the sequencing of resistant strains versus susceptible ones (73, 74). Although antibiotic treatments often led to global changes in gene expression (75, 76), very few genes enriched with mutations were found when bacterial cells were exposed to certain antibiotics (16, 18). Here, we determined a subset of specific genes with a high number of mutations under rifampicin selection, including some specific genes that could be associated with drug resistance and/or adaptative responses to the antibiotic. Some interesting candidates related to cell permeability (*trkB* and *mchK*) seemed to be potential genes associated with low to intermediate levels of drug resistance, as previously reported (29, 30). Interestingly, nonsynonymous mutations in homologs of some candidate genes have been detected in rifampicin-resistant isolates of M. tuberculosis: Rv3200c (MSMEI_1903; *mchK*), Rv1384 (MSMEI_2973; *carB*), Rv2006 (MSMEI_3863; *otsB1*), Rv2343c (MSMEI_4371; *dnaG*), Rv3823c (MSMEI_4625; *mmpL*), Rv0803 (MSMEI_5666; *purL*), and Rv3689 (MSMEI_6055; transmembrane protein gene) (in parentheses is the homologous gene in M. smegmatis) (77). Further site-specific modification of the candidate genes would be required to confirm the association of specific mutations with rifampicin resistance.

In conclusion, MA evolution in combination with WGS constitutes an effective approach to study the total pool of genome-wide mutations upon antibiotic selection in mycobacteria. This strategy enabled us to evaluate the emergence of drug resistance mutations, the mechanisms and evolutionary pathways associated with drug resistance, and the potential mutagenic effect of rifampicin. Finally, the appearance of genomic mutations under antibiotic selective pressure could be studied in pathogenic mycobacteria such as M. tuberculosis, to elucidate the impact of antibiotic treatments on the emergence and evolution of drug-resistant isolates.

## MATERIALS AND METHODS

### Bacterial strains and media.

M. smegmatis wild-type strain mc^2^ 155 (American Type Culture Collection; 700084) and its noncanonical MMR-deficient (Δ*nucS* mutant) derivative (21) were grown at 37°C in Middlebrook 7H9 broth or 7H10 agar (Difco) with 0.5% glycerol and Tween 80 (0.05% in 7H10 agar and 0.5% in 7H9 broth). The growth of bacterial strains in liquid medium was carried out in a shaker incubator at 250 rpm at 37°C. Rifampicin (Rifaldin; Sanofi) was dissolved in sterile distilled water and added to 7H10 agar plates as required.

### MA experiments.

MA experiments were performed following a previously described procedure (17). However, in this study MA experiments were performed in the presence of increasing concentrations of rifampicin. In this work, 20 independent cell lines were generated from the wild-type strain mc^2^ 155 and the same number from its isogenic Δ*nucS* derivative. These lines were independently evolved for 40 weeks (280 days) in parallel. Bacteria were initially streaked onto Middlebrook 7H10 plates supplemented with rifampicin at a starting concentration of 0.25 μg mL^−1^. Every week, a single random colony from each line was transferred to a new plate. The concentration of rifampicin was doubled every 5 weeks (five serial passages for each antibiotic concentration), ending the evolution at week 40 with a final concentration of 32 μg mL^−1^. The evolved lines generated at weeks 10, 20, 25, 30, 35, and 40 were stored at −80°C for further analysis.

### Genomic DNA preparation and WGS analysis.

Since most of the MA lines did not progress at the final rifampicin concentration of the evolution, we sequenced all evolved MA lines obtained at week 35 with 16 μg mL^−1^ rifampicin (38 lines, including 18 wild-type-derived lines and 20 Δ*nucS* mutant-derived lines, numbered 1 to 20). The two parental strains (named mc^2^ and Δ*nucS* 0) were also sequenced as references. Genomic DNA was extracted from each of the 40 strains following the standard protocol for preparation of high-quality mycobacterial genomic DNA, as previously described (78). The integrity of each DNA sample and the absence of RNA contamination were confirmed by DNA agarose gel electrophoresis, while its concentration and purity were measured using a NanoDrop-2000 spectrophotometer (Thermo Fisher Scientific) and a Qubit 3.0 fluorometer (Life Technologies). WGS libraries were constructed with the NEBNext Ultra DNA library preparation kit (Illumina, San Diego, CA). Sequencing was performed on the Illumina MiSeq instrument using a MiSeq v.2 sequencing kit to obtain 250-bp paired-end reads.

### Bioinformatics analyses to detect SNPs and indels.

WGS data were processed according to the following procedure. First, raw fastq files were filtered and trimmed with fastp (v.0.12.5) using the following parameters: –cut_by_quality3 –cut_window_size = 10 –cut_mean_quality = 20 –length_required = 50 –correction. Filtered fastq files were mapped against the M. smegmatis mc^2^ 155 reference genome NC_018289.1 with the bwa mem algorithm (v.0.7.12-r1039). Only reads that mapped the reference genome with a mapping quality equal to 60 were retained, and duplicates were removed using picard tools (v.2.18.0). Samtools (v.1.3.1) was used to obtain a pileup file, which was later scanned for genomic variants with VarScan2 (v.2.3.7). For SNP calling, we applied the following filters: minimum depth of 10 reads at the genomic position, the presence of SNPs in a minimum of 6 reads, minimum average quality of 25, and the presence of SNPs in both strands and at a minimum frequency of 0.5. Indels were called with gatk (v.3.8-1-0-gf15c1c3ef), using the HaplotypeCaller program, and filtered with the VariantFiltration module with the filters recommended for strict filtering: LowQd QD < 2.0, HighFS FS > 200.0, HighSOR SOR > 3.0, Low40MQ MQ < 40.0, LowReadPRS ReadPosRankSum 20.0, and LowDepth DP < 20. All detected variants were annotated with SnpEff (v.4.2). We analyzed the SNPs and indels detected in the evolved MA lines by comparison with their corresponding parental strain, wild type or Δ*nucS* mutant, as appropriate. The set of variants found in the parental strains with respect to the M. smegmatis reference genome, detected in this study and in our previous work (17), were filtered using in-house Perl scripts and not considered for subsequent analyses. In this filtering step, SNPs having an indel in close proximity (10 nucleotides [nt] upstream or downstream) were removed. In the case of indels, those with low depth (DP < 10) were also discarded from subsequent analyses.

### Calculation of mutation rates.

The mutation rate per genome was calculated from WGS data by dividing the number of mutations by the total estimated generations (Table 1), and the mutation rate per nucleotide was calculated by dividing the number of mutations by the number of generations and the total sequenced sites in each genome (Table 1; see Tables S1 and S2 in the supplemental material). These calculations were performed for the overall mutations and for each specific type of change in the DNA. The number of generations was estimated as previously described (17). First, we established a relationship between the number of viable cells present in a colony (*N*) and its area, as described by Lee et al. (79). Then, wild-type and Δ*nucS* MA lines were streaked on plates supplemented with each concentration used in the experimental evolution and the diameter of the colonies was measured after 7 days to estimate their number of viable cells. The total number of generations of each line was calculated as the sum of the generations per passage (*n*), being *n* = log_2_ *N*. Statistical analyses (*t* test) were performed to compare the mutation rates for each type of mutation in the presence and absence of rifampicin (Table S5).

### Sequencing of *rpoB* gene of the MA lines.

To identify *rpoB* mutations, the full-length *rpoB* gene was sequenced from all of the lines during experimental evolution (weeks 0, 10, 20, 25, 30, 35, and 40). In each case, M. smegmatis *rpoB* was amplified by PCR using the set of primers indicated in Table S9. Five different overlapping PCR fragments were generated to cover the full-length *rpoB* gene in each sample. PCR products were sequenced using the Sanger method, and the sequences were aligned with an *rpoB* wild-type sequence to identify mutations.

### Sequencing of *trkB* and *mchK* genes of the MA lines.

MA lines with mutations in *trkB* and *mchK* at week 35 of evolution were identified from WGS data. The *trkB* and *mchK* genes were then sequenced in the same MA lines from the previous weeks to determine when each mutation emerged. The *trkB* and *mchK* genes were amplified by PCR using the primers indicated in Table S9, and PCR products were sequenced using the Sanger method.

### Determination of rifampicin MICs.

The rifampicin MICs of parental strains were evaluated by Etest on solid media. The rifampicin MICs of the MA lines stored at the different weeks of evolution were determined by the broth microdilution method following the guidelines of the Clinical and Laboratory Standards Institute (CLSI) and using resazurin for the detection of mycobacterial growth as previously reported (80). Serial 2-fold dilutions of rifampicin were performed in 100 μL of Middlebrook 7H9 broth in round-bottom 96-well plates. All wells were then inoculated with 5 μL of overnight cultures of the M. smegmatis MA lines, previously diluted 1:100 (final dilution of ~1:2,000). The plates were incubated at 37°C for 48 to 72 h. Then, 30 μL of resazurin solution (200 μg mL^−1^) was added to all of the wells, and plates were incubated overnight at 37°C. The MIC was defined as the lowest drug concentration that prevented the change of the resazurin color from blue (oxidized form) to pink (reduced form), indicating the inhibition of the bacterial growth.

### Statistical analyses.

Standard statistical analyses were performed using the rstatix (v.0.7.0) and gmodels (v.2.18.1.1) packages of R (v.4.0.5).

The *dN*/*dS* ratio was calculated by using the formula (nonsynonymous variants/nonsynonymous sites)/(synonymous variants/synonymous sites). The potential synonymous and nonsynonymous substitution sites for each gene were calculated using the SNAP method (81), available at https://www.hiv.lanl.gov/content/sequence/SNAP/perlsnap.html (82).

### Data accessibility.

Raw FASTQ files from WGS of this work, in the presence of rifampicin, and the previous MA experiment, in the absence of antibiotic (17), have been deposited into the Sequence Read Archive (SRA) database under BioProject accession no. PRJNA899888 (this work) and PRJNA898800 (17).
