# Supplementary material for: Selective Pressure by Rifampicin Modulates Mutation Rates and Evolutionary Trajectories of Mycobacterial Genomes
Source: Microbiol Spectr. 2023 Jul 12;11(4):e01017-23. doi: 10.1128/spectrum.01017-23 (PMC10433840; doi:10.1128/spectrum.01017-23)
Supplement: Supplemental file 1 — Supplemental material. Download spectrum.01017-23-s0001.pdf, PDF file, 0.2 MB [file spectrum.01017-23-s0001.pdf]

A

trkB mutations						
Week	10	20	25	30	35	40
[Rif] (µg/ml)	0.5	2	4	8	16	32
Line						
Δ*nucS* 5	A130A	A130A	A130A	A130A	A130A	A130A
Δ*nucS* 12	NO	NO	NO	NO	W111R	extinct
Δ*nucS* 18	NO	V91A	V91A	V91A	V91A	V91A
Δ*nucS* 20	NO	NO	NO	G12D	G12D	G12D

B

mchK mutations						
Week	10	20	25	30	35	40
[Rif] (µg/ml)	0.5	2	4	8	16	32
Line						
mc^2 4	NO	NO	NO	NO	Δ - 1c	extinct
mc^2 6	NO	NO	NO	T163I	T163I	extinct
mc^2 19	NO	NO	NO	A216V	A216V	extinct
Δ*nucS* 1	NO	NO	NO	R120Q	R120Q	R120Q
Δ*nucS* 2	NO	NO	NO	NO	E316G	E316G
Δ*nucS* 8	NO	NO	NO	P321L	P321L	extinct
Δ*nucS* 9	NO	NO	V203A	V203A	V203A	V203A
Δ*nucS* 19	NO	NO	Q265R	Q265R	Q265R	Q265R

Rif MIC (µg ml^{-1}) — 1 - 2 No resistant; 4 - 16 Low; 32 - 128 Intermediate; 256 - 1024 High

Figure S1. Mutations in *trkB* and *mchK* in the MA lines. A. Emergence of *trkB* mutations in the MA lines. **B**. Emergence of *mchK* mutations in the MA lines. Tables show the appearance of mutations in *trkB* and *mchK* in each MA line during experimental evolution (in weeks). Only MA lines with mutations in these genes are represented. Levels of rifampicin resistance of the evolved lines are indicated according to their MICs values, with the following color code: no resistance, 1 – 2 µg ml^{-1} (grey); low, 4 – 16 µg ml^{-1} (light red); intermediate, 32 – 128 µg ml^{-1} (medium red); and high, 256 – 1024 µg ml^{-1} (dark red).
