# Supplementary material for: Selective Pressure by Rifampicin Modulates Mutation Rates and Evolutionary Trajectories of Mycobacterial Genomes
Source: Microbiol Spectr. 2023 Jul 12;11(4):e01017-23. doi: 10.1128/spectrum.01017-23 (PMC10433840; doi:10.1128/spectrum.01017-23)
Supplement: Supplemental file 2 — Supplemental material. Download spectrum.01017-23-s0002.pdf, PDF file, 0.1 MB [file spectrum.01017-23-s0002.pdf]

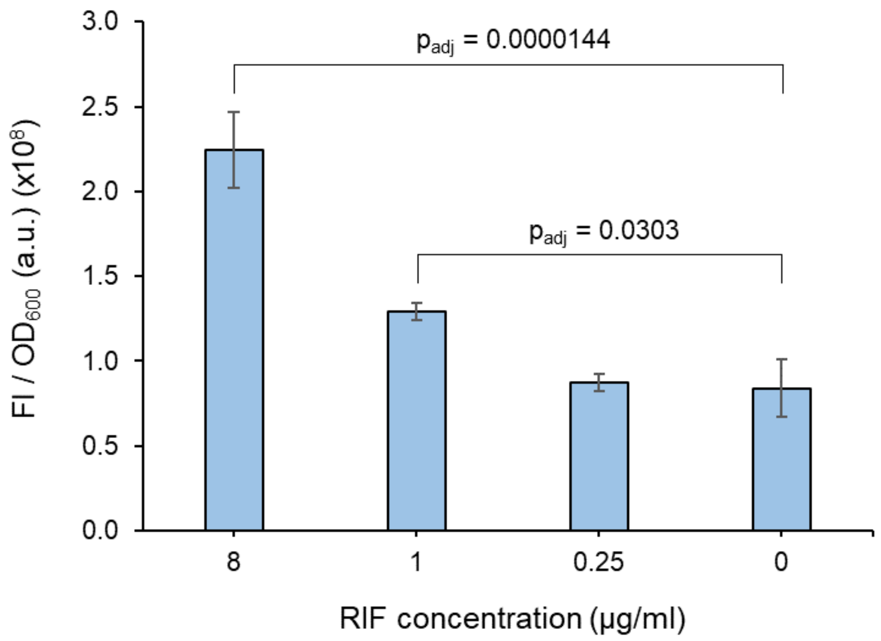

Supplementary Figure S2. ROS levels of *M. smegmatis* under rifampicin exposure. ROS levels were measured using 2′,7′-dichlorofluorescin diacetate, DCFDA (Merck), a cell-permeable non-fluorescent probe that turns fluorescent upon oxidation. *M. smegmatis* cells at exponential phase were treated with rifampicin (0.25, 1 and 8 µg/ml) for 3 h (*M. smegmatis* generation time). Cultures without rifampicin treatment (0 µg/ml) were used as negative control. Cells were pelleted by centrifugation and resuspended in saline solution with and without DCFDA (10 µM). Controls without the probe were also included to monitor autofluorescence. After the addition of the probe, cells were incubated for 15 min at 37 ºC and fluorescence intensity (excitation 488 nm and emission 530 nm) was measured in a VICTOR® Nivo plate reader (Perkin Elmer) using ViewPlate-96 Black plates (Perkin Elmer). Bars represent the mean of fluorescence intensity (FI) levels normalized by OD_{600} measurements (in arbitrary units, a. u.) of three independent cultures. Fluorescence of the cells under the different treatments but without the probe was subtracted. Error bars indicate standard deviation (SD). Adjusted p-value is shown for significant comparisons with untreated cells (0 µg/ml) (pairwise t-test with Bonferroni correction).
