# Supplementary material for: Selective Pressure by Rifampicin Modulates Mutation Rates and Evolutionary Trajectories of Mycobacterial Genomes
Source: Microbiol Spectr. 2023 Jul 12;11(4):e01017-23. doi: 10.1128/spectrum.01017-23 (PMC10433840; doi:10.1128/spectrum.01017-23)
Supplement: Supplemental file 5 — Supplemental material. Download spectrum.01017-23-s0005.pdf, PDF file, 0.1 MB [file spectrum.01017-23-s0005.pdf]

Table S8. Mutation rates identified in this experiment excluding the mutations from genes shown in Table 2.

	mc^2 155 (WT)			Δ*nucS*		
	**No. lines**	**Total generations**	**Generations per line**	**No. lines**	**Total generations**	**Generations per line**
	18	16,308	906	20	18,315	916
**Mutation type**	**No. mutations**	**Mutation rate per genome per generation (x10^{-3})***	**Mutation rate per nucleotide per generation (x10^{-10})***	**No. mutations**	**Mutation rate per genome per generation (x10^{-3})***	**Mutation rate per nucleotide per generation (x10^{-10})***
**Total**	132	8.09 ± 1.38	12.01 ± 2.04	3,101	169.36 ± 10.61	251.66 ± 15.83
**BPSs**	95	5.83 ± 1.24	8.65 ± 1.83	3,062	167.24 ± 10.44	248.52 ± 15.58
**Indels**	37	2.26± 0.80	3.36 ± 1.18	39	2.12 ± 0.66	3.15 ± 0.98

* The mutation rate per genome or nucleotide per generation ± 95% CI (confidence intervals) is shown.
