# Supplementary material for: Selective Pressure by Rifampicin Modulates Mutation Rates and Evolutionary Trajectories of Mycobacterial Genomes
Source: Microbiol Spectr. 2023 Jul 12;11(4):e01017-23. doi: 10.1128/spectrum.01017-23 (PMC10433840; doi:10.1128/spectrum.01017-23)
Supplement: Supplemental file 6 — Supplemental material. Download spectrum.01017-23-s0006.pdf, PDF file, 0.1 MB [file spectrum.01017-23-s0006.pdf]

Table S9. Primers used in this study

Primer name*	Sequence	Target gene	Source
M.smegmatis-rpoB-FWD (f)	GCTGATCCAGAACCAGATCC	*rpoB* (MSMEI_1328, MSMEG_1367)	(*16*)
M.smegmatis-rpoB-REV (r)	GATGACACCGGTCTTGTCG	*rpoB* (MSMEI_1328, MSMEG_1367)	(*16*)
rpoB-F1 (f)	TGTCGTTGCGTCCAGGGTTCTGGA	*rpoB* (MSMEI_1328, MSMEG_1367)	This study
rpoB-R1 (r)	GCTCCACGATCTGCTCGTTGGTCC	*rpoB* (MSMEI_1328, MSMEG_1367)	This study
rpoB-F2 (f)	GTATCGACCGCAAGCGCCGCCAGC	*rpoB* (MSMEI_1328, MSMEG_1367)	This study
rpoB-R2 (r)	CGAACCGATCAGACCGATGTTGGG	*rpoB* (MSMEI_1328, MSMEG_1367)	This study
rpoB-F3 (f)	GACGTGCACCCCAGCCACTACGGC	*rpoB* (MSMEI_1328, MSMEG_1367)	This study
rpoB-R3 (r)	AGCTTGGTGTCGCGGGCATCGATC	*rpoB* (MSMEI_1328, MSMEG_1367)	This study
rpoB-F4 (f)	GAACCGCCTGGTCGAAGAGGACGT	*rpoB* (MSMEI_1328, MSMEG_1367)	This study
rpoB-R4 (r)	ACATGTAGCCAACCGTCACCGGGT	*rpoB* (MSMEI_1328, MSMEG_1367)	This study
rpoB-F5 (f)	ACGCCGACGGCAAGGCGACGCTGT	*rpoB* (MSMEI_1328, MSMEG_1367)	This study
rpoB-R5 (r)	CTACGCGAGATCCTCGACGGACGC	*rpoB* (MSMEI_1328, MSMEG_1367)	This study
TrkB2769seqF (f)	ATGAAAGTCGCCATCGCCGGTGCC	*trkB* (MSMEI_2701, MSMEG_2769)	This study
TrkB2769seqR (r)	CTAACGCCGCGTGGGCCGCAGCAG	*trkB* (MSMEI_2701, MSMEG_2769)	This study
MchK1945seqF (f)	GTGGCTAAAGGCAGGTTACGGCGC	*mchK* (MSMEI_1903, MSMEG_1945)	This study
MchK1945seqR (r)	TCATCGTTCGGCGTCCGCACTGCG	*mchK* (MSMEI_1903, MSMEG_1945)	This study

* (f): forward, (r): reverse.
